# microclass: an R-package for 16S taxonomy classification

**DOI:** 10.1186/s12859-017-1583-2

**Published:** 2017-03-16

**Authors:** Kristian Hovde Liland, Hilde Vinje, Lars Snipen

**Affiliations:** 1 Department of Chemistry, Biotechnology and Food Sciences, Norwegian University of Life Sciences, Ås, P.O. Box 5003, N-1432 Norway; 2 Nofima - Norwegian Institute of Food, Fisheries and Aquaculture Research, Osloveien 1, Ås, N-1430 Norway

**Keywords:** R, Taxonomy, 16S

## Abstract

**Background:**

Taxonomic classification based on the 16S rRNA gene sequence is important for the profiling of microbial communities. In addition to giving the best possible accuracy, it is also important to quantify uncertainties in the classifications.

**Results:**

We present an R package with tools for making such classifications, where the heavy computations are implemented in C++ but operated through the standard R interface. The user may train classifiers based on specialized data sets, but we also supply a ready-to-use function trained on a comprehensive training data set designed specifically for this purpose. This tool also includes some novel ways to quantify uncertainties in the classifications.

**Conclusions:**

Based on input sequences of varying length and quality, we demonstrate how the output from the classifications can be used to obtain high quality taxonomic assignments from 16S sequences within the R computing environment. The package is publicly available at the Comprehensive R Archive Network.

**Electronic supplementary material:**

The online version of this article (doi:10.1186/s12859-017-1583-2) contains supplementary material, which is available to authorized users.

## Background

The profiling of microbial communities by the sequencing of the 16S rRNA gene has become a standard approach in metagenomics [1]. This means that collected DNA is subject to a targeted sequencing to extract a selected region of the 16S gene from all organisms in the sample. The actual content of the sample can then be described by performing a large scale taxonomic classification of these sequences, i.e. assign them to the proper taxonomic bin, also referred to as binning [2]. Since 16S-based microbial profiling has become such a widely adopted approach, it is also important that the bioinformatics tools involved are optimized to the highest standard. A widely used tool for this job is the RDP-classifier [3]. It is beyond doubt a good tool for this job, but at the same time it is not perfect, and in a systematic testing of this and other approaches we found there were always other methods that performed better [4]. Alternative tools are a benefit to the scientific community, and here we present a software to be used within the popular R computing environment [5].

There are some issues that must be considered when it comes to making tools for the binning of 16S sequences. First, the pattern recognition algorithm itself must be capable of recognizing the, sometimes small, differences in DNA that separates taxa. It must also handle the huge amount of bins or categories we are facing here, thousands rather than 2–3 which is often the case in textbook literature. Precision also very much depends on the quality of the training data [6]. Due to the ever expanding taxonomy of prokaryotes, there are no comprehensive gold standard data sets available. Along with the microclass package described here, we also supply the microcontax R data package, containing designed data sets based on a consensus taxonomy assignment among several data repositories. This is probably the closest we get to a gold standard today.

Speed is another issue. With today’s sequencing technology and low prices, a data set may easily contain millions of reads. Some procedures for OTU (Operational Taxonomic Unit) picking will start out by classifying reads to pre-defined taxa [7]. Thus, the number of sequences to classify may be huge. Other procedures cluster the reads before taxonomy assignments, defining OTU’s as ’spherical’ clusters in a space of evolutionary distances approximated by alignment percentage identity, and then classify only the cluster centroids [8]. In some applications, e.g. in forensic applications [9], we are more interested in recognizing specific taxonomic profiles rather than discovering new taxa. In such cases the classification of all reads into pre-defined bins is clearly what we seek.

Uncertainty is the third issue. In any collection of reads there will be a number of sequences that cannot be given a high-confidence classification. There are several reasons for this. First, the taxonomy itself is not always well defined, and sometimes even high-quality sequences fall on the border between existing taxa, making the classification uncertain. Second, due to sequencing errors and chimeras some reads may be difficult to recognize, and third, some microbial communities will contain new taxa not previously seen.

In the presented R-package we have implemented some algorithms that have proved efficient and/or are much used for 16S taxonomic classification. Efforts have been made to make them both fast and memory-efficient. All methods can be trained on new data, but we have also supplied the package with a ready-to-use tool that is already trained and optimized for the contax.trim data set from [10]. This tool also quantifies uncertainties in a new way. The microclass R-package, as well as its symbiotic data package microcontax, are freely available at the Comprehensive R Archive Network (CRAN, [11]).

## Implementation

### The multinomial method

Based on our previous testing of *K*-mer based classification methods in [4] we found that the best overall results were obtained by the algorithm denoted the multinomial method [12]. Thus, we have focused the attention on this method in this package. The function multinomTrain is used to train a model of this type on any data set containing FASTA-formatted sequences along with the correct taxon assignments for each sequence. The function multinomClassify is then used to classify new sequences based on a trained model.

Both training of a multinomial model and classification of new sequences involves counting a large number of *K*-mers (overlapping words of length *K*) in the sequences. The overhead when doing such operations is large, and efficient vectorization is difficult to achieve. A direct implementation would also require the computation of a matrix product of size (*N*×4^*K*^) · (4^*K*^×*M*), where *N* and *M* are the number of sequences to classify and the number of taxa in the training data, respectively. This is a time consuming task for large *N*, *M* and *K*. Therefore, these computations have been implemented in C++ through the Rcpp [13] interface in R, and some short-cuts are made, which will be explained in the following paragraphs.

The nucleotide sequences are first converted to integer vectors my mapping A, C, G and T (or U) to 0, 1, 2, and 3, while all other letters are mapped to - 4^15^. The latter is done to easily discard *K*-mers including alien symbols when counting. For training of a multinomial model, all *K*-mers of each taxon are counted. The counting itself is done by sliding a window along the integer vector of each sequence and computing a position as the inner product between [ 4^*K*−1^,4^*K*−2^,…,4,1] and the integers in the window. For each of the inner products, this position in the taxon’s counting vector is increased by 1. The result is a matrix, ***X***, of size (*M*×4^*K*^) that holds the counts for all *K*-mers in all taxa. Finally, each position in the matrix is re-scaled to $\log _{2}\left (\frac {x_{ij}-P/4^{K}}{\sum {x_{i\cdot }}-P}\right)$, where *P* is the number of pseudo-counts added. This is stored in an (*M*×4^*K*^) matrix named ***Q*** to represent multinomial log-probabilities with pseudo-counts.

When classifying new sequences using the multinomial method, we avoid the mentioned matrix product by combining the *K*-mer counting with summing of multinomial log-probabilities. For each counted *K*-mer, the corresponding column in the ***Q*** matrix is added to the result, thus never explicitly creating the *K*-mer count matrix or performing the product with ***Q***. As such we reduce from (4^*K*^·*M*) operations to ((*n*−*K*)·*M*) for a new sequence of length *n*. For full 16S sequences (with *n*≈1500 bases) the number of calculations will be lower for *K*>5.27 and is easily parallelized.

### The taxMachine

Users often want a ready-to-use tool to classify (many) 16S sequences without having to perform all the training. Based on the work in [4] we have arrived at an optimized tool for classifying 16S sequences, called taxMachine in this package. The taxMachine is based on using the multinomial method with a word length of *K*=8 and a pseudo count of 100. It has been trained on full-length 16S sequences to recognize full or partial (reads) sequences at the genus level, using the designed and optimized contax.trim data set for training. See the microcontax data package for details. The taxMachine includes computations of classification uncertainties that requires a detailed explanation.

### Classification uncertainty

Uncertainty in a taxonomic classification can be split into two types. The first type is when a sequence happens to be very close to the decision boundary between two or more taxa. We can be fairly certain it belongs to one of these taxa, but it lacks the final discriminative power to safely assign it to one of them. The second type of uncertainty occurs when something completely new is seen. This is not uncommon in metagenome samples, and should be flagged separately since it may indicate sequencing errors, chimeras or some novel type of organism.

#### The *d*-score

**Fig. 1:**
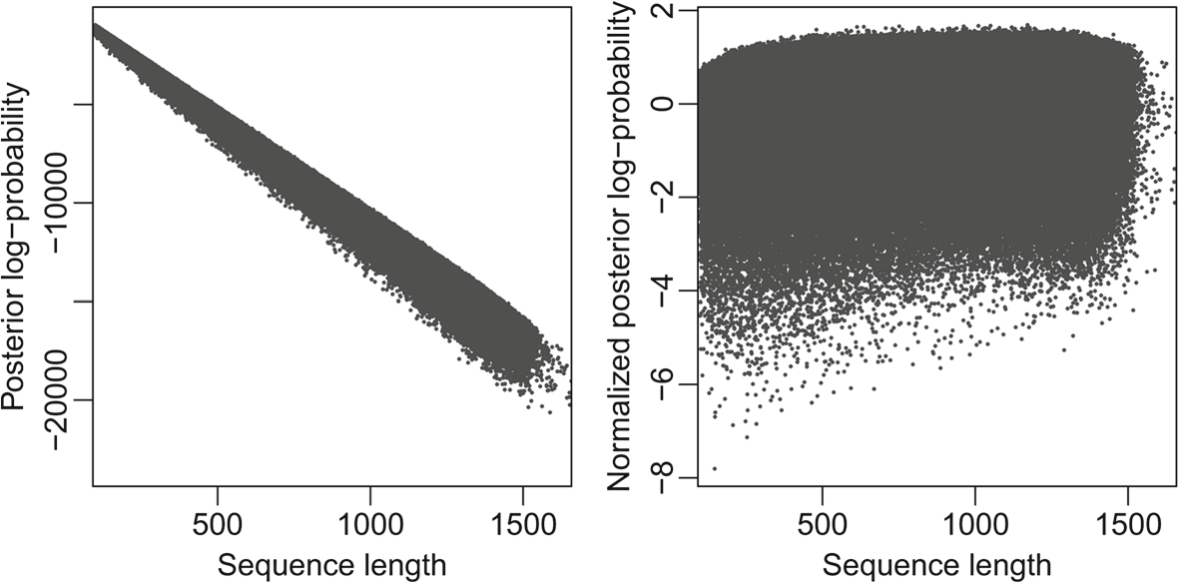
Posterior log-probability normalization. The *left panel* shows posterior log-probabilities for 38 781 sequences. The sequences are random sub-sequences of the contax.trim data set, spanning all lengths from 100 bases to more than 1500. Every sequence has been classified using the multinomial model trained on the full-length data, and each *dot marks* the maximum posterior log-probability for one sequence. There is clearly a linear trend in the values, with larger variance for longer sequences. In the *right panel* the same values are plotted after the normalization procedure described in the text

The first type of uncertainty is measured by what we name the *d*-score. Consider sequence *i* in a set of sequences that we want to classify. In the taxMachine the predicted genus of sequence *i* is found by computing the posterior log-probability for every genus, and classifying to the genus with maximum value. If we sort all posterior log-probabilities for sequence *i* in descending order, *p*_*i*,1_ denotes this maximum, while *p*_*i*,2_ is the second largest, etc. These log-probabilities all depend on the sequence length, since a longer sequence will in general contain more unique *K*-mers, and the posterior log-probability will be a sum with more (negative) terms. This is illustrated in the left panel of Fig. 1. Here we have sampled fragments of random length (>100 bases) from all sequences in the contax.trim data set, and then classified them, collecting the *p*_*i*,1_ for sequence *i*=1,…,38 781. The *p*_*i*,1_ values are clearly biased by sequence length, and their variance is also increasing for longer sequences.

We first normalize the posterior log-probability with respect to sequence length. We fitted linear regression models describing how both the mean and the standard deviation of the data in the left panel of Fig. 1 varies by sequence length *l*. Thus, if sequence *i* has length *l* it gets the normalized posterior log-probability

1 $$ \tilde{p}_{i,1} = \frac{p_{i,1}-\hat{p}_{l}}{\hat{s}_{l}}   $$

where $\hat {p}_{l}$ and $\hat {s}_{l}$ are the predicted mean and standard deviation at sequence length *l*, using the fitted regression models. Note that *p*_*i*,2_ (and any other posterior log-probability) can be normalized in the same way, using the same fitted regression model.

The *d*-score of sequence *i* is simply the difference between the largest and the second largest normalized posterior log-probability:

2 $$ d_{i} = \tilde{p}_{i,1} - \tilde{p}_{i,2}  $$

If we are near a decision boundary we expect *d*_*i*_≈0 since the second best genus is almost as good as the best. On the other hand, if *d*_*i*_>>0 it means the predicted genus is much more likely than any other, and we have a high confidence classification.

#### The *r*-score

The second type of uncertainty is high if we see something very different from what we have in the training data set. Consider sequence *i* belonging to genus *g* with corresponding normalized maximum posterior log-probability $\tilde {p}_{i,1}$ from (1). From all sequences belonging to genus *g* we computed the sample mean and sample standard deviation of the $\tilde {p}_{i,1}$’s, denoted $\bar {p}_{g}$ and *s*_*g*_ respectively. The *r*-score for sequence *i* is the standardized residual

3 $$ r_{i} = \frac{\tilde{p}_{i,1} - \bar{p}_{g}}{\bar{s}_{g}}   $$

where $\bar {s}_{g}$ is a smoothed version of *s*_*g*_ as explained below. Thus, the r-score is a standardized measure of how different a sequence is from its predicted genus centre.

**Fig. 2:**
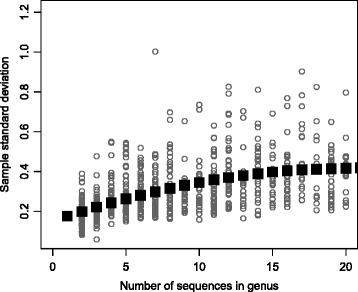
Smoothing genus standard deviation. The sample standard deviations for every genus (*grey rings*) are plotted against genus size (number of sequences). The *black squares* are the mean values for each genus size, after loess-smoothing as described in the text

Different genera have different sequence diversity, which is reflected in different values of the sample standard deviation *s*_*g*_. However, many genera have too few sequences to provide a reliable estimate of this standard deviation, some even have only 1 sequence making *s*_*g*_ impossible to compute. Thus, the $\bar {s}_{g}$ in (3) is based on a simple smoothing. First, all sample standard deviations where grouped by genus-size. In Fig. 2 we show how smaller genera (few sequences) tend to have smaller sample standard deviations. We used the loess method [14] to estimate the size-specific sample standard deviation, shown as black squares in Fig. 2. We denote this *s*_*n*_ where *n* is the genus-size. If genus *g* has size *n* we get the genus-specific standard deviation estimate as

4 $$ \bar{s}_{g} = \sqrt{\frac{(n - 1) s_{g}^{2} + s_{n}^{2}}{n}}   $$

When a new sequence is classified, we do not know its true genus. The predicted genus is then used as a plug-in in (3), i.e. we use $\bar {p}_{g}$ and $\bar {s}_{g}$ where *g* is the predicted genus. If the resulting *r*_*i*_ has a large negative value, it means the computed $\tilde {p}_{i,1}$ is much smaller than the average $\bar {p}_{g}$ for genus *g*, and sequence *i* is unlikely to belong to this genus even if this is where it maximizes the posterior probability.

Exactly how negative is the *r*-score for an un-recognized sequence? To guide this decision we computed the *r*-scores for all sequences in the contax.full data set [10], and from this we computed the empirical cumulative distribution function. For any given *r*_*i*_ value this gives us the probability of having an *r*-score this small, or smaller, given that the sequence was from the training data. A very small probability means the sequence is very unusual compared to the training data.

### Other methods

The package also contains some alternatives to the multinomial method, mostly for comparisons. The RDP-classifier [3] is a popular tool used in many metagenome applications. The version implemented here is a stripped version without the bootstrapping effort to quantify uncertainties in the classifications. It has been implemented in C++ and accelerated similarly to the multinomial method, see above for details.

A classification using BLAST is also included, since this approach has been common. It is both slower and less precise then the other methods. It requires the BLAST+ software to be installed on the system.

## Results and discussion

The microclass package provides optimized tools for taxonomic classification of 16S sequence data in the R computing environment. Some well established and proven methods are available to all users of R, with the possibility to train all methods on new and specialized data sets. However, a ready-made classification tool, taxMachine, is also supplied as an R-function. This has been optimized in several ways to produce the most accurate classifications at the genus level, without consuming too much memory. Specifically, it employs *K*-mers of length 8, where an increase to *K*=9 or *K*=10 comes at high cost in computation time and memory consumption compared to the small gain in accuracy for genus classifications. Pseudo counts have been set to 100 in the taxMachine as a robust compromise regardless of sequence length (see Additional file 1: Figure S1).

The classification of 16S is the most fundamental approach to profiling a microbial community, and due to the explosion in metagenomic research activities, tools for recognizing taxa from 16S sequences (reads) should be tuned to their optimal performance. The taxMachine R-function builds on a parallelized sparse-matrix implementation of the multinomial method that makes it efficient both with respect to speed and memory usage. It has been trained on the contax.trim data set, containing 38 871 full-length high-quality sequences covering 1774 genera, where all sequences have a consensus taxonomy, making it the closest we get to a well-balanced gold standard training set.

The proposed implementation of *K*-mer counting simply discards a word if it contains an ambiguous character. The main reason for this is the added overhead to the computations by introducing another layer of logic to handle these symbols. For instance the occurrence of the letter D in a sequence means that the base in question could be a G, A or T. One could add 1/3 count to each of the three resulting words, but this would require a substantially slower *K*-mer counting logic. Since informative ambiguous characters (not N) are rarely seen in reads, we chose to disregard these words and keep the speed advantage of the integer logic.

As described in the Implementation section the taxMachine provides information about classification uncertainty, based on the posterior probabilities of the multinomial model. The very first step needed in these computations is to remove the bias from sequence length in the log-probabilities, as suggested in Eq. (1). The right panel of Fig. 1 shows how the normalized posterior log-probabilities have no apparent trends over sequence length, as opposed to the raw-values in the left panel. This normalization makes it possible to compute uncertainty/reliability scores to sequences regardless of their exact lengths.

The proposed *d*-score for a sequence is the difference in score between the most likely and the second most likely taxon. A *d*-score close to 0 means the sequence is close to a decision border, being almost equally similar to both taxa, and more likely to be mis-classified. To visualize this, we classified fragments of all sequences in the contax.trim data set using the taxMachine. We considered fragments of typical read-lengths; 120–150 and 270–300 bases, which is typical for Illumina HiSeq and MiSeq raw data, and 450–500 bases, which is typical for Roche 454 and merged (paired-end) Illumina MiSeq data. From each of the original 38 871 sequences we sampled 10 such fragments at random locations along each sequence.

**Fig. 3:**
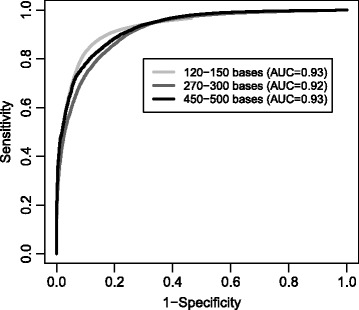
ROC analysis of *d*-scores. Based on the classification of read-length fragments, each sequence was either correctly or incorrectly classified. Each sequence also has a *d*-score. Ranking by *d*-score produced a separation of incorrect and correct classifications as indicated by the ROC-curves and the corresponding AUC statistics. Each curve is based on results for 387 810 sequences

**Fig. 4:**
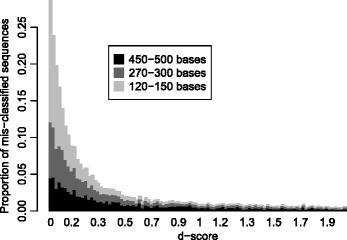
Histogram of *d*-scores. The histograms show the proportions of *d*-scores for the mis-classified sequences only, in the range from 0 to 2.0. This is from the same results as Fig. 3, with three different read-lengths

Comparing the predicted genus to the assigned genus, the error percentages were 1% for 450–500 bases reads, 3% for 270–300 bases and 11% for 120–150 bases, respectively, when the sequences from which the reads were generated were included in the model training (see Additional file 2: Table S1 for cross-validated success rates). The *d*-score should ideally be small for the mis-classified sequences, and large for the others. In Fig. 3 we show a ROC analysis where all sequences are ranked by their *d*-score. Based on the large AUC statistics (0.92−0.93) we conclude that a small *d*-score is an effective criterion for identifying mis-classified sequences. In Fig. 4 we show how the *d*-score distributes for the mis-classified sequences. Clearly, the majority has a *d*-score below 1.0 and the shorter the reads the more the *d*-scores are concentrated near 0. The probability of mis-classification will in general never exceed that of correct classification even for *d*-score almost at 0, but at 0 there is a 50−50 chance of making a mistake. Various applications will require different strictness, but a classification with *d*-score above 1.0 can in general be considered safe. Based on the results in Fig. 4 we found that among all classifications with a *d*>1 there were 1.1, 0.7 and 0.3% errors for input sequences of lengths 120–150, 270–300 and 450–500 bases, respectively.

**Fig. 5:**
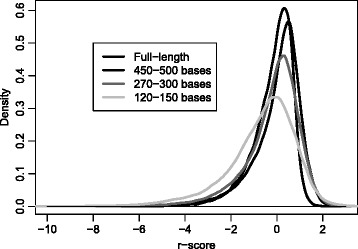
Densities of *r*-scores. Based only on correctly classified sequences, the densities show how the *r*-scores distribute. The densities were estimated by a non-parametric kernel smoother in R. Only negative *r*-scores are of interest, since a (very) negative value indicates a (very) unusual sequence

We face a different type of uncertainty when we collect sequences very different from what we have seen in the training data set. In the Implementation section we describe the *r*-score to detect this. A negative *r*-score means the sequence has a lower probability than average for the assigned taxon. But how much lower than the average is critical? To investigate this we used the same results as mentioned above, classifying sub-sequences of typical read-lengths, but in addition we also included full-length sequences. We then computed the *r*-score for all correctly classified sequences. Figure 5 shows the *r*-score densities for the various cases. It is the heavy left tail of the densities that is of interest. First, we notice there is some difference between densities for sequences of different lengths. Next, we see that even for correctly classified sequences, a very negative *r*-score occurs in a few cases. An *r*-score below −4 to −5 is rare for correctly classified sequences, and indicates an unusual sequence. The taxMachine also provides a probabilistic measure related to the *r*-score. Based on the contax.full data set (664 199 sequences) we computed densities similar to those in Fig. 5, and from these the empirical cumulative distribution functions. The probability *Pr*(*r*<*r*_*i*_|trainingdata) is found from this distribution, for any given *r*_*i*_. This probability reflects how unusual a sequence is compared to the training data, and if this is very small, its classification is not reliable.

**Fig. 6:**
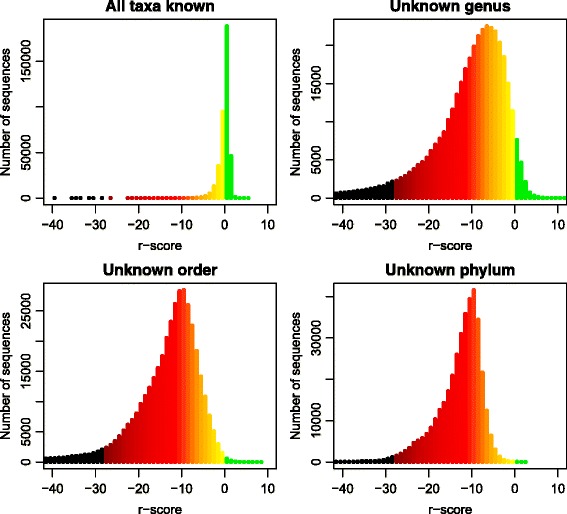
Effect of unknown taxa on *r*-scores. The four histograms show distribution of *r*-scores. The colors are: *Green* for all positive *r*-scores and *black* for scores more negative than ever observed in the contax.full data set. The transition from *yellow to red* indicates gradually smaller probabilities (from around 10^−1^ at *yellow* to 10^−8^ at *dark red*) of observing the corresponding *r*-score in the training set. *Red colors* are probabilities below 10^−5^. The *upper left panel* are *r*-scores where all classified taxa are present in the training data, i.e. no unknown taxa. In the *upper right panel* each genus is unknown, i.e. when classifying a sequence from genus A, there are no sequences from this genus in the training data. In the *lower panels* the same procedure has been repeated but the training data lack sequences from the same order and phylum, respectively

In Fig. 6 we demonstrate how the *r*-score histograms change when faced with sequences from unknown taxa. Here we have only focused on sub-sequences of lengths 450−500, but the results were similar for other sequence lengths as well. We used a taxon-wise cross-validation, i.e. in each iteration we leave out all sequences from a taxon, train the model on the rest, and classify the sequences of the left-out taxon. This means all classified sequences are from an unknown taxon, not part of the training data. The upper left panel shows, for comparison, how the distribution looks like without this cross-validation (mean *r*-score −0.1). In the upper right panel each genus has been left out, i.e. the training data contains no sequences from the genus of the classified sequence. The *r*-scores in general become more negative even if some are still quite large, even positive (mean *r*-score −13.5). This is not surprising, since many genera are quite similar, and a sequence from the neighboring genus may not look very unusual. In the lower panels we have cross-validated over order and phylum (mean *r*-scores −17.0 and −19.5), making the classified sequences gradually more distant from those of the training data. The lower left tail of the histograms seems thinner, but a substantial number of sequences got very negative *r*-scores well outside the range of the plots. The proportion of sequences in the green-yellow region (large *r*-scores) is gradually smaller.

**Fig. 7:**
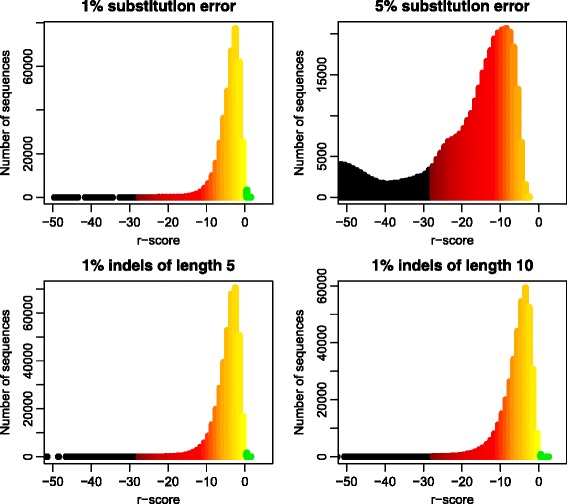
Effect of sequencing error. Histograms of *r*-scores similar to those in Fig. 6. Reads of lengths 450−500 bases were corrupted at random before classification. In the *upper panels* 1*%* and 5*%* of the bases in each read were corrupted with a base different from the correct one. In the *lower panels* insertions and/or deletions of lengths 5 and 10 bases were distributed randomly at 1*%* of the positions in each read

Figure 7 illustrates, in a similar way, the effect of sequencing errors. The sequences from the upper-left panel of Fig. 6 have been corrupted with random substitution errors at two levels, and then classified. The results are seen in the upper panels of Fig. 7. A 1*%* substitution error level will distort the *r*-scores, but still the majority of sequences are recognized to an acceptable level, with *r*-scores above −6. In total, more than 98*%* of the sequences are correctly classified. At 5*%* substitution error the majority of the reads have *r*-scores well into the red and even black region, indicating unrecognised sequences. Still, more than 90*%* of them are correctly classified, mostly those with the larger *r*-scores. With NGS technologies like Illumina or PacBio (circular consensus), the substitution error rate is usually well below 1*%* [15, 16]. In the lower panels of Fig. 7 we have corrupted the sequences by insertions and/or deletions in a similar way. For illumina reads indels are virtually non-existing [15], while for PacBio reads they occur at the same rate as substitutions [16]. We see that even with insertions or deletions of length 10, the effect on the R-score is small. The classification accuracy is around 98*%* for both length 5 and 10 indels, and only slightly smaller than for 1*%* substitution error. This is as expected, since *K*-mer methods like the ones we have here benefit from having the sequencing errors concentrated as a few indels rather then many substitution errors scattered at random along the sequence. We conclude that classifications of the taxMachine is largely unaffected by the sequencing error levels we expect from current NGS technology.

Chimera sequences will also result in sequences that are different in *K*-mer composition from its source sequences. In Additional file 3: Figure S2 we show an example of such a mixture, including *d*- and *r*-scores.

The *r*-score, and/or its corresponding probability, may be used to discard sequences that appear unusual. As always, the strictness of this procedure will depend on the application. For most applications we would not discard reads unless they are in the lower 1*%* or 0.1*%* quantile, at least (probabilities smaller than 10^−2^−10^−3^). Instead of fixing some threshold, and discarding reads, one may also use these probabilities as weights, and give reads with small *r*-scores less weight. When tabulating read-counts into a taxonomic profile, this seems like a natural procedure. Conservative estimates of the expected success rates in classifying new reads and full sequences can be found in Additional file 2: Table S1.

The heavy computations of the microclass package are performed in optimized, parallelized C++. This means that the users can comfortably work in R, knowing that reasonably large data can be processed on a personal computer. Larger problems can be tackled in a further parallelized fashion on computational clusters, simply by splitting data into blocks for separate processing. As the package has an open GPL 2/3 licence, reuse of the code in other, possibly pure C++, implementations is allowed as long as the licencing is correct and proper acknowledgements are used.

## Conclusions

The package microclass offers tools for taxonomic classification based on 16S rRNA sequence data to the R community. There are function for training classifiers on your own, specialised data sets, and for using these classifiers to classify new sequences. The taxMachine function has synthesized the designed training data from the microcontax data package with the methods of this package, and is our suggested tool for general classifications. It also implements some novel ways to express uncertainties in the classifications, indicating if the input sequences are difficult to recognize.

## Additional files

Additional file 1 Supplementary Figure 1 Effect of pseudo-counts. The fraction of mis-classified sequences after 10-fold cross-validation, using various number of pseudo-counts in the training of the multinomial models (horizontal axes). This is based on the contax.trim data set, and models have been trained at all levels of the taxonomy, from domain to genus (panels). The effects of different choices of pseudo-counts are modest, and at the genus-level the use of 100 pseudo-counts is a reasonable compromise for all types of input sequence lengths. The amplicon sequences are obtained by using the primer pair 515*F* (GTGYCAGCMGCCGCGGTAA) and 806*rB* (GTGYCAGCMGCCGCGGTAA) to extract subsequences, in general matching the V3-V4 region of the 16S gene. (PDF 5 kb)

Additional file 2 Supplementary Table 1 Performance of the multinomial classifier. A table with results describing the performance of the multinomial classifier. (PDF 14 kb)

Additional file 3 Supplementary Figure 2 Chimera example. We constructed a chimera sequence by mixing *Salmonella* and *Enterococcus*. Both sequences have 1503 bases, and the chimera starts as *Salmonella* and ends as *Enterococcus*. The horizontal axis shows the number of *Salmonella* bases, i.e. if the *n* first bases are *Salmonella* then the 1503−*n* last bases are *Enterococcus*. The blue axis/dots shows how the *d*-score changes as we gradually mix the two sequences, and the tan axis/dots similar for the *r*-score. The red/yellow/green band at the top shows the classification at each chimera level. On the left side, when only a minority of the sequence is *Salmonella*, it is recognized as *Enterococcus* (green region). In the middle, it is misclassified as *Escherichia* (yellow region), which is a fairly close relative of *Salmonella*, but as the *Salmonella*-part gets majority it is recognized as *Salmonella* (red region). Notice the low *d*-score values in the middle section, indicating uncertain classifications. The *r*-scores also drop in the middle region. The ’jumps’ in *r*-score are due to the dependency of the classified genus. The posterior log-probabilities do not change abruptly, but the *r*-score is related to what we expect for the assigned genus, and the latter causes the switches. (PDF 37 kb)

## Abbreviations

BLAST: Basic local alignment search tool
CRAN: Comprehensive R archive network
OTU: Operational taxonomic unit
RDP: Ribosomal database project
