# Supplementary figures and images for: microclass: an R-package for 16S taxonomy classification
Source: BMC Bioinformatics. 2017 Mar 16;18:172. doi: 10.1186/s12859-017-1583-2 (PMC5353803; doi:10.1186/s12859-017-1583-2)

domain

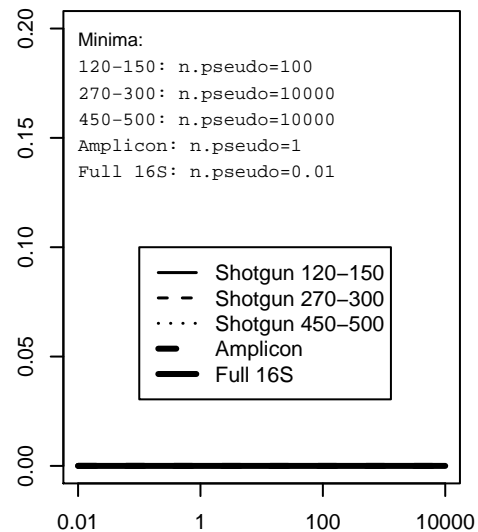

phylum

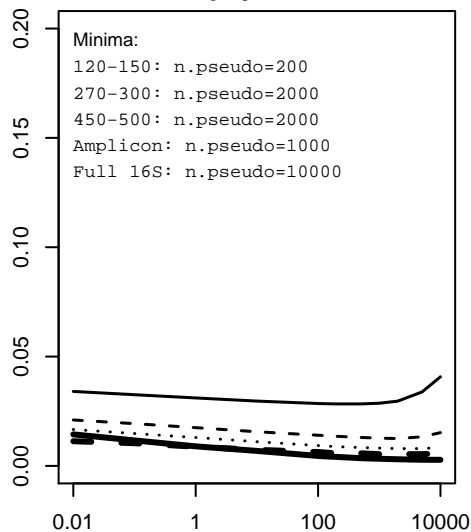

class

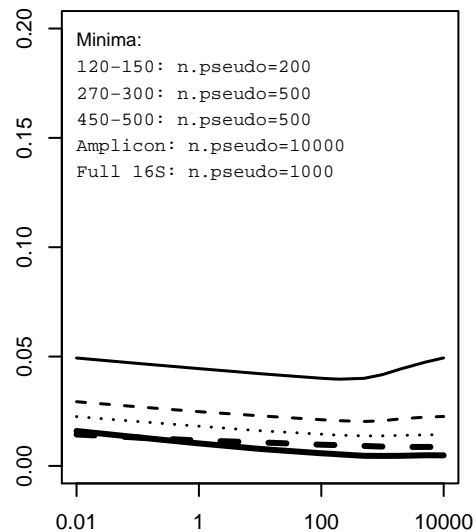

order

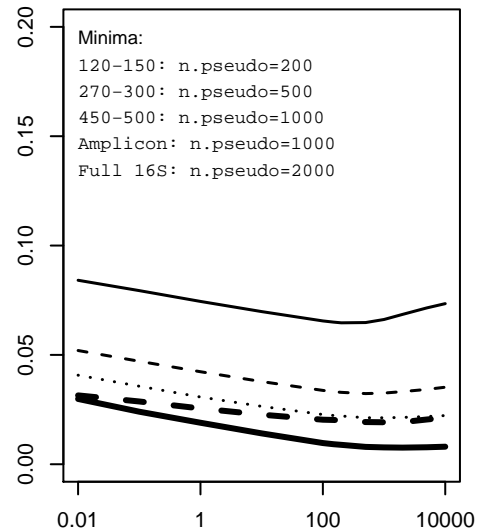

family

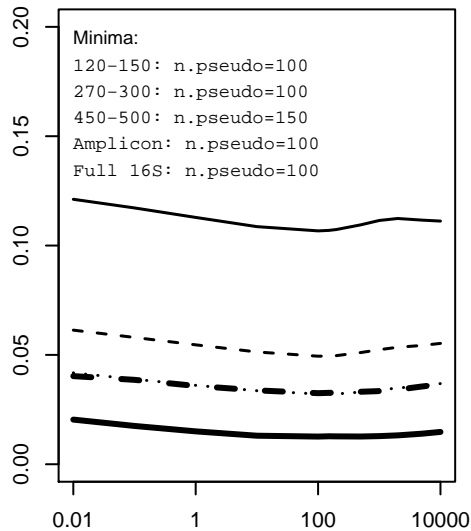

genus

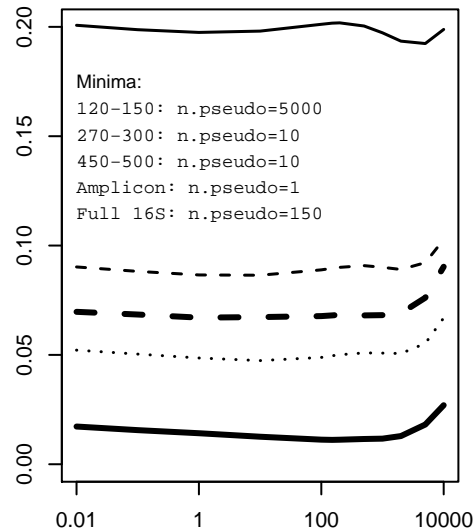

Supplement: Additional file 1 — Supplementary Figure 1 Effect of pseudo-counts. The fraction of mis-classified sequences after 10-fold cross-validation, using various number of pseudo-counts in the training of the multinomial models (horizontal axes). This is based on the contax.trim data set, and models have been trained at all levels of the taxonomy, from domain to genus (panels). The effects of different choices of pseudo-counts are modest, and at the genus-level the use of 100 pseudo-counts is a reasonable compromise for all types of input sequence lengths. The amplicon sequences are obtained by using the primer pair 515F (GTGYCAGCMGCCGCGGTAA) and 806rB (GTGYCAGCMGCCGCGGTAA) to extract subsequences, in general matching the V3-V4 region of the 16S gene. (PDF 5 kb) [file 12859_2017_1583_MOESM1_ESM.pdf]

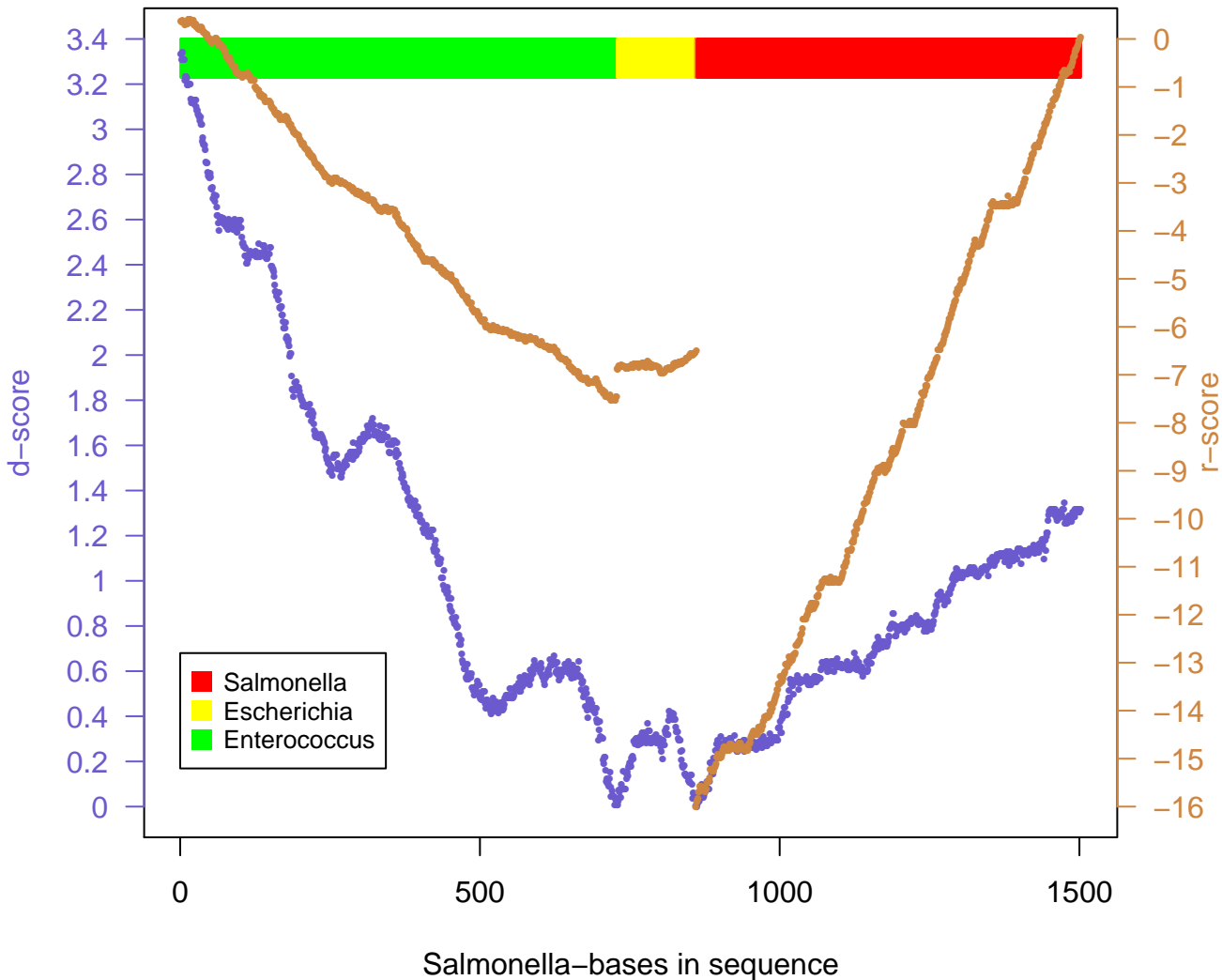

Supplement: Additional file 3 — Supplementary Figure 2 Chimera example. We constructed a chimera sequence by mixing Salmonella and Enterococcus. Both sequences have 1503 bases, and the chimera starts as Salmonella and ends as Enterococcus. The horizontal axis shows the number of Salmonella bases, i.e. if the n first bases are Salmonella then the 1503−n last bases are Enterococcus. The blue axis/dots shows how the d-score changes as we gradually mix the two sequences, and the tan axis/dots similar for the r-score. The red/yellow/green band at the top shows the classification at each chimera level. On the left side, when only a minority of the sequence is Salmonella, it is recognized as Enterococcus (green region). In the middle, it is misclassified as Escherichia (yellow region), which is a fairly close relative of Salmonella, but as the Salmonella-part gets majority it is recognized as Salmonella (red region). Notice the low d-score values in the middle section, indicating uncertain classifications. The r-scores also drop in the middle region. The ’jumps’ in r-score are due to the dependency of the classified genus. The posterior log-probabilities do not change abruptly, but the r-score is related to what we expect for the assigned genus, and the latter causes the switches. (PDF 37 kb) [file 12859_2017_1583_MOESM3_ESM.pdf]
