# Supplementary material for: microclass: an R-package for 16S taxonomy classification
Source: BMC Bioinformatics. 2017 Mar 16;18:172. doi: 10.1186/s12859-017-1583-2 (PMC5353803; doi:10.1186/s12859-017-1583-2)
Supplement: Additional file 2 — Supplementary Table 1 Performance of the multinomial classifier. A table with results describing the performance of the multinomial classifier. (PDF 14 kb) [file 12859_2017_1583_MOESM2_ESM.pdf]

Supplementary Table 1 - Performance of the multinomial classifier

Table 1: Number of misclassified for every 1000 sequences classified in the `contax.trim` data set using 10-fold cross-validation, removal of singletons, $K = 8$ and $n.pseudo = 100$. In parentheses are the effects of changing from $K = 8$ to $K = 10$, i.e. the reduction in mis-classified sequences. Increasing K leads to a substantial increase in memory usage and computing time, as the problem grows by $O(4^K)$, and at the genus-level the gain of increasing beyond $K = 8$ is too small.

	120-150bp	270-300bp	450-500bp	515f+806rB	Full 16S
domain	0 (-0)	0 (-0)	0 (-0)	0 (-0)	0 (-0)
phylum	29(-11)	14 (-4)	9 (-3)	6 (-4)	5 (-3)
class	40 (-8)	21 (-4)	14 (-3)	10 (-5)	6 (-3)
order	65 (-9)	34 (-6)	23 (-6)	20 (-7)	10 (-6)
family	107 (-3)	49 (-5)	32 (-5)	32 (-4)	12 (-5)
genus	197(+1)	85 (-0)	45 (-1)	64 (-2)	7 (-0)
